# Early detection and correction of preoperative anemia in patients undergoing colorectal surgery—a prospective study

**DOI:** 10.1007/s10151-025-03131-5

**Published:** 2025-04-05

**Authors:** A. de Wit, B. T. Bootsma, D. E. Huisman, G. Kazemier, F. Daams

**Affiliations:** 1 Department of Surgery, Amsterdam University Medical Centers, De Boelelaan 1117, 1081 HV Amsterdam, The Netherlands; 2 Cancer Center Amsterdam, Amsterdam, The Netherlands

**Keywords:** Anemia, Iron transfusion, Colorectal surgery, Anastomotic leakage

## Abstract

**Introduction:**

Preoperative anemia is an important target in preventing colorectal anastomotic leakage (CAL). However, it is not consistently detected and corrected in patients undergoing colorectal surgery. This study aimed to evaluate the impact of early detection and correction of preoperative anemia on perioperative outcomes and CAL.

**Methods:**

This was a prospective subanalysis of an international open-labeled trial, which implemented an enhanced care bundle to prevent CAL after elective colorectal surgeries. It introduced interventions for early detection and correction of preoperative anemia. Primary outcome was the incidence of preoperative anemia and the effect of early correction. Secondary outcomes included the impact on CAL, postoperative course, and mortality.

**Results:**

The study included 899 patients across eight European hospitals (September 2021–December 2023). Preoperative anemia was identified in 35.0% (*n* = 315) of participants, with 77.4% (*n* = 192) receiving iron therapy. Hemoglobin levels decreased in 4.2% (*n* = 13), remained stable in 45.8% (*n* = 143), and increased in 50.0% (*n* = 156) (*p* < 0.001). Perioperative hyperglycemia was more common among patients with anemia (7.8% versus 16.4%, *p* < 0.001). CAL occurred in 6.1% (*n* = 53) of patients. Anemia correction and changes in hemoglobin levels after iron treatment were not significantly associated with CAL, other complications, or mortality.

**Conclusions:**

Early detection and correction of preoperative anemia is achievable. However, routine preoperative administration of iron alone, without concurrently optimizing other CAL risk factors, does not result in CAL prevention. Preoperative anemia indicates overall poor physiological fitness rather than being an isolated risk factor.

**Trial number:**

NCT05250882 (20-01-2022).

## Introduction

Colorectal anastomotic leakage (CAL) is a severe complication, occurring in 2–19% of patients undergoing colorectal surgery [1]. Given that CAL can lead to abdominal sepsis, necessity for stoma formation, increased mortality, and heightened hospital costs, various strategies have been proposed to prevent this dreadful complication [2, 3]. Over time, the focus has shifted toward identifying modifiable risk factors for CAL to develop the most effective preventive strategies [4].

Perioperative anemia has been identified as the most significant modifiable risk factor for CAL in a large international trial (OR 5.4, *p* < 0.001) [5]. Although some studies reported conflicting results, the majority demonstrated a significant association between preoperative anemia and CAL incidence [6, 7]. The prevalence of preoperative anemia varies between 22% and 67%, depending on the specific definition of anemia and hemoglobin cutoff values used [8, 9]. Anemia is particularly prevalent in colorectal cancer patients, with an increased incidence of 40–80%, primarily due to intraluminal blood loss from the tumor site [10].

Preoperative anemia is predominantly caused by iron deficiency, which arises from impaired iron absorption, reduced iron availability, nutrient deficiencies, and occult intraluminal blood loss [11]. This leads to a reduced production of hemoglobin. The shortage of circulating hemoglobin impairs perfusion and oxygenation at the newly constructed anastomosis, both of which are critical for tissue healing, thereby increasing the risk of CAL [12].

Despite evidence indicating that preoperative anemia is a significant predictor of CAL and an important target for optimization, it is not consistently detected and corrected during the preoperative phase. Furthermore, a standardized care pathway for optimization in patients undergoing colorectal surgery is not available. The recent DoubleCheck study was the first to implement an enhanced care bundle, which consisted of early detection and correction of preoperative anemia, among other interventions [13]. The aim of this study was to obtain detailed information on the impact of early detection and correction of preoperative anemia on perioperative and postoperative outcomes in patients undergoing colorectal surgery.

## Methods

### Study design

This open-labeled interventional study was conducted between September 2021 and December 2023 across eight European hospitals. It was designed as a prospective subanalysis of the DoubleCheck study [13]. Ethical approval was obtained from the Ethical Committee of the Vrije Universiteit and all participating hospitals, and declared exempt from the Medical Research Involving Human Subjects Act (WMO). The study was performed in accordance with ethical standards as laid down in the 1964 Declaration of Helsinki. The study protocol was registered in the Clinical Trials Registry (clinicaltrials.gov; NCT05250882). All patients provided written informed consent prior to participation.

### Participants

All patients scheduled for elective colorectal surgery involving anastomosis creation were eligible for inclusion. Patients were included regardless of whether the indication for resection was benign or malignant, and construction of a diverting stoma was permitted. Exclusion criteria included age under 18 years, emergency surgery, and inability to comprehend the informed consent. All patients received treatment according to the complete DoubleCheck enhanced care bundle [13].

### Outcomes

The primary outcome of this study was the incidence of preoperative anemia following the implementation of active screening in accordance with the enhanced care bundle. Additionally, the study examined the effect of preoperative anemia correction and compliance with the enhanced care bundle. Anemia was defined as a blood hemoglobin level < 7.5 mmol/L (12.1 g/dL) for female patients and < 8.0 mmol/L (12.9 g/dL) for male patients, combined with a ferritin < 30.0 μg/L or the specified hemoglobin levels with ferritin between 30.0 μg/L and 100.0 μg/L, transferrin saturation < 15.0–20.0%, and C-reactive protein > 5.0 mg/L [14, 15]. Anemia severity was classified as severe (< 6.0 mmol/L; < 9.7 g/dL), mild (6.0–7.0 mmol/L; 9.7–11.3 g/dL), and minor (> 7.0 mmol/L; > 11.3 g/dL). Changes in hemoglobin levels were categorized as decreased when levels dropped by > 0.5 mmol/L, increased when elevated by > 0.5 mmol/L, and unchanged when within the range of −0.5 and 0.5 mmol/L.

Secondary outcomes included the effects of active preoperative screening and anemia correction on CAL, as well as postoperative outcomes and mortality. These secondary outcomes were collected at 30 and 90 days postoperatively. CAL was defined using Reisinger’s criteria and categorized according to both the International Study Group of Rectal Cancer (ISREC) and Clavien–Dindo (CD) classifications [16–18]. Other postoperative complications, including ileus, delayed gastric emptying, pneumonia, wound infection, bleeding, and atrium fibrillation, were determined and classified according to the CD classification.

### Intervention

Preoperative blood tests, including hemoglobin, ferritin, transferrin saturation, and CRP, were performed during the patient’s initial visit at the outpatient clinic. If abnormalities were detected, the patient was referred for anemia correction. Intravenous ferric (III) carboxymaltose was recommended for treatment, although the final decision between intravenous or oral iron supplementation was left to the discretion of the clinician [15, 19]. After iron treatment, the hemoglobin level was reassessed at an unspecified time prior to hospital admission. Additionally, a hemoglobin measurement was conducted during the perioperative time-out moment in all patients. While there were no strict guidelines for postoperative hemoglobin monitoring, it was recommended to determine the hemoglobin level at least once before hospital discharge. Table 1 provides detailed recommendations for anemia detection and treatment.

**Table 1:** Anemia detection and correction

	Timing	Cutoff values	Action
Preoperative phase			
Outpatient clinic	Always (Hb, Fe, Tr-Sat, CRP)	Hb ♀ < 7.5 mmol/L + Fe < 30 μg/L Hb ♂ < 8 mmol/L + Fe < 30 μg/L OR CRP > 5 + Tsat < 20% + Fe 30–100 μg/L	Administration ferric (III) carboxymaltose (preferably intravenous)
Hospital admission	If anemic at outpatient clinic (Hb)	♀ < 7.5 mmol/L; ♂ < 8 mmol/L	–
Perioperative phase			
Anastomosis creation	Always (Hb)	♀ < 7.5 mmol/L; ♂ < 8 mmol/L	Transfusion according to local protocol
Postoperative phase			
POD1	If anemic at outpatient clinic (Hb) OR > 500 ml blood loss (Hb)	According to local protocol	Transfusion according to local protocol
Before discharge	Recommended (Hb)	According to local protocol	–

*Hb* hemoglobin, *Fe* iron, *Tr-Sat* transferrin saturation, *CRP* c-reactive protein, *POD1* postoperative day one

The DoubleCheck enhanced care bundle was implemented in patients undergoing colorectal surgery [13]. The bundle included anemia correction, hyperglycemia detection, maintenance of normothermia, correct timing of antibiotic prophylaxis, avoidance of unnecessary vasopressors administration, and abstention from epidural analgesia. Compliance with these interventions was assessed during an additional perioperative time-out moment, just prior to the creation of the anastomosis. At all times, clinicians were allowed to modify any intervention to ensue patient safety. Additional preoperative and perioperative safety measures, such as bowel decontamination, stapler-doughnut inspection, air-leak testing, Indocyanine Green (ICG), and endoscopy, were routinely performed, although practices varied across hospitals. The Enhanced Recovery After Surgery (ERAS)-guidelines were fully implemented before the study start [20].

Patient-related (age, sex, American Society of Anestesiologists (ASA)-score, body mass index (BMI), comorbidities, intoxications, pathology, detection by screening program, distance from anal verge, neoadjuvant therapy) and surgery-related (blood loss, blood transfusion, oxygen saturation, mean arterial pressure, urine output, fluid administration, surgery duration, surgical procedure, surgical approach, intraoperative events, additional procedures, stoma placement) baseline characteristics were extracted from patient files. Furthermore, information regarding the postoperative course (length of stay, intensive care unit stay, readmission, re-interventions, complications) and mortality were collected. Additionally, data on pre-, peri- and postoperative anemia presence, progression of related blood measurements, and treatment were also gathered in this manner.

### Statistical analysis

The collected data were analyzed using Statistical Package for the Social Sciences software (SPSS version 28.0, Chicago, IL, USA). Patients with missing blood levels necessary for anemia diagnosis were excluded from the analysis. Descriptive statistics were used to examine differences in baseline variables and study outcomes, and subsequent subgroup analysis. Categorical variables were presented as proportions (%) and analyzed using a Pearson’s chi-squared test or analysis of variance (ANOVA) test. Continuous variables were reported as means (standard deviation) or medians (interquartile range), and analyzed using a Student’s *t*-test or Mann–Whitney *U* test, depending on data skewness. Additional univariate and multivariate regression analyses were conducted to further delineate differences among study groups. Outcomes were displayed as odds ratio’s (OR), 95% confidence intervals (95% CI), and *p*-values. A *p*-value of < 0.05 was considered statistically significant.

## Results

### Baseline characteristics

Detailed information on the detection and correction of anemia was available for 899 patients (99.7%). The study population was 54.2% male (*n* = 487), with a median age of 68 years (18–92 years). Of the patients, 15.1% (*n* = 122) underwent surgery for benign indications, while 84.9% (*n* = 686) for malignant indications. The surgical procedures performed included right-sided hemicolectomy in 39.7% (*n* = 357) of the cases, ileocoecal resection in 1.9% (*n* = 17), transversal resection in 2.4% (*n* = 22), left-sided hemicolectomy in 8.7% (*n* = 78), sigmoidectomy in 26.1% (*n* = 235), total mesorectal excision (TME) or low aneterior resection (LAR) in 16.2% (*n* = 146), transanal total mesorectal excision (TaTME) in 1.1% (*n* = 10), and other procedures in 3.8% (*n* = 34). Additionally, 3.6% (*n* = 32) received a diverting stoma (1.6% colon; 12.2% rectum).

### Preoperative anemia

Preoperative hemoglobin and ferritin levels were measured in 97.1% (*n* = 873) of the patients. The mean hemoglobin level was 7.9 mmol/L (12.7 g/dL). The incidence of anemia at the initial outpatient clinic visit was 35.0% (*n* = 315), which was not statistically different in male patients compared with female patients (34.3% versus 38.2%, *p* = 0.235). Severe preoperative anemia was present in 29.5% (*n* = 93) of the patients, mild in 35.2% (*n* = 111), and minor in 35.2% (*n* = 111).

Table 2 presents the baseline characteristics of patients with and without anemia. Patients with preoperative anemia were significantly older (67 versus 74 years, *p* < 0.001), had a higher ASA score (2.1 versus 2.4, *p* < 0.001), and were more frequently diabetic (8.3% versus 18.9%, *p* < 0.001). Additionally, the performed surgical procedures differed significantly between the two groups (*p* < 0.001), and preoperative anemia was more prevalent in patients with malignant diseases (81.9% versus 90.7%, *p* < 0.001). The mean blood loss (85 versus 101 ml, *p* < 0.001) and number of perioperative blood transfusions (0.4% versus 3.2%, *p* < 0.001) were significantly higher in the anemic group. Perioperative hyperglycemia was more common among patients with anemia (7.8% versus 16.4%, *p* < 0.001), while the other DoubleCheck risk factors hypothermia (*p* = 0.973), incorrect antibiotic prophylaxis (*p* = 0.753), vasopressors (*p* = 0.314), and epidural analgesia (*p* = 0.109) did not differ significantly between the anemic and non-anemic groups.

**Table 2:** Baseline characteristics: preoperative anemia

	No anemia (*n* = 558)	Anemia (*n* = 315)	*p*-value
Preoperative			
Age (years)	67 (16)	74 (16)	** < 0.001**
Sex (male)	55.9% (312)	51.7% (163)	0.235
ASA score	2 (0)	2 (1)	** < 0.001**
BMI (kg/m^2^)	25.8 (5)	25.4 (5)	0.474
Diabetes mellitus (yes)	8.3% (45)	18.9% (58)	** < 0.001**
Pathology (malign)	81.9% (406)	90.7% (262)	** < 0.001**
Neoadjuvant therapy (yes)	88.7% (401)	93.4% (269)	**0.004**
Intoxications			
Current smoker (yes)	11.0% (61)	7.4% (23)	0.197
> 14 packyears (yes)	44.4% (84)	41.9% (44)	0.674
Alcohol (yes)	59.8% (317)	46.2% (140)	** < 0.001**
> 4 units per day (yes)	1.4% (4)	0.8% (1)	0.640
Drugs (yes)	1.3% (7)	0	0.054
Perioperative			
Procedure			
Right-sided hemicolectomy	26.2% (146)	54.9% (173)	** < 0.001**
Extended right-sided hemicolectomy	3.2% (18)	4.4% (14)	
Ileocecal resection	1.8% (10)	1.9% (6)	
Transversum resection	1.6% (9)	3.5% (11)	
Left-sided hemicolectomy	9.5% (53)	7.6% (24)	
LAR/TME	21.0% (117)	7.3% (23)	
TaTME	1.1% (6)	0.6% (2)	
Sigmoidectomy	30.8% (172)	17.8% (56)	
Other	4.9% (27)	1.9% (6)	
Surgery duration (min)	142 (56)	150 (60)	0.309
Surgical approach (open)	1.3% (7)	1.9% (6)	0.562
Blood loss (ml)	50 (80)	50 (130)	** < 0.001**
Intraoperative blood transfusion (yes)	0.4% (2)	3.2% (10)	** < 0.001**
Intraoperative event (yes)	4.6% (25)	6.4% (20)	0.255
Stoma (yes)	3.0% (17)	4.4% (14)	0.284
Additional organ resection (yes)	21.4% (117)	23.2% (72)	0.559
Hyperglycemia (yes)	7.8% (42)	16.4% (49)	** < 0.001**
Hypothermia (yes)	54.4% (301)	54.3% (170)	0.973
Incorrect antibiotic prophylaxis (yes)	1.1% (6)	1.3% (4)	0.735
Vasopressors (yes)	60.0% (335)	63.5% (200)	0.314
Epidural (yes)	2.7% (15)	4.8% (15)	0.109

Categorical variables are expressed as proportions (%) and continuous variables as medians (interquartile range)

*p*-values < 0.05 were considered statistically significant and marked bold

### Preoperative anemia correction

Following the diagnosis of anemia, 77.4% (*n* = 192) of patients were treated according to the enhanced care bundle. Among these, 87.9% (*n* = 167) received intravenous iron transfusions, while 12.1% (*n* = 23) were given oral suppletion in the weeks prior to surgery. In the treatment group, hemoglobin levels decreased in 4.2% (*n* = 13) of patients, remained unchanged in 45.8% (*n* = 143), and increased in 50.0% (*n* = 156). The mean hemoglobin level during creation of the anastomosis was 8.2 mmol/L (13.2 g/dL). Perioperative anemia was observed in 32.3% (*n* = 290) of the patients. A severe preoperative anemia was present in 6.2% (*n* = 18) of the patients, mild in 37.6% (*n* = 109), and minor in 56.2% (*n* = 163), which was significantly different from preoperative measurements (*p* < 0.001) (Table 3).

**Table 3:** Preoperative anemia correction

Blood measurements			
	Preoperative	Perioperative	*p*-value
Hemoglobin (mmol/L)	7.9 (3.1–11.4)	8.2 (4.2–12.5)	** < 0.001**
Anemia (yes)	35.0% (315)	32.3% (290)	** < 0.001**
Severe (< 6 mmol/L)	29.5% (93)	6.2% (18)	** < 0.001**
Mild (6–7 mmol/L)	35.2% (111)	37.6% (109)	
Minor (> 7 mmol/L)	35.2% (111)	56.2% (163)	

Correction of preoperative anemia			
From anemic to non-anemic	23.4% (73)		
Change in hemoglobin level			
Unchanged (−0.5 to 0.5 mmol/L)	45.8% (143)		
Declined (> 0.5 mmol/L)	4.2% (13)		
Increased (> 0.5 mmol/L)	50.0% (156)		

Categorical variables are expressed as proportions (%) and continuous variables as medians (range)

*p*-values < 0.05 were considered statistically significant and marked bold

Table 4 outlines differences between the preoperative iron treatment and non-treatment group. Age, sex, and comorbidities were not significantly different between the two groups. Patients who received preoperative anemia correction underwent significantly different surgical procedures (*p* = 0.041) compared with those who did not. Moreover, in the treatment group, exposure to hypothermia (66.1% versus 50.5%, *p* = 0.040) was significantly lower, while vasopressor administration (48.2% versus 72.4%, *p* < 0.001) was significantly more frequent.

**Table 4:** Baseline characteristics: preoperative correction anemia

	Not corrected (*n* = 56)	Corrected (*n* = 192)	*p*-Value
Preoperative			
Age (years)	73 (16)	75 (15)	0.246
Sex (male)	55.4% (31)	48.4% (93)	0.362
ASA score	2 (1)	2 (1)	0.210
BMI (kg/m^2^)	25.4 (5)	25.6 (5)	0.734
Diabetes mellitus (yes)	10.7% (6)	21.5% (41)	0.071
Pathology (malign)	86.8% (46)	93.8% (165)	0.141
Neoadjuvant therapy (yes)	12.0% (6)	4.5% (8)	0.316
Intoxications			
Current smoker (yes)	3.6% (2)	7.4% (14)	0.472
> 14 packyears (yes)	42.9% (6)	50.7% (35)	0.591
Alcohol (yes)	48.1% (25)	48.7% (91)	0.940
> 4 units per day (yes)	4.3% (1)	1.2% (1)	0.392
Drugs (yes)	0	0	–
Perioperative			
Procedure			
Right-sided hemicolectomy	48.2% (27)	63.5% (122)	**0.041**
Extended right-sided hemicolectomy	1.8% (1)	5.2% (10)	
Ileocecal resection	1.8% (1)	0.5% (1)	
Transversum resection	5.4% (3)	2.6% (5)	
Left-sided hemicolectomy	1.8% (1)	6.3% (12)	
LAR/TME	8.9% (5)	7.8% (15)	
TaTME	0	1.0% (2)	
Sigmoidectomy	28.6% (16)	10.9% (21)	
Other	3.6% (2)	2.1% (4)	
Surgery duration (min)	150 (79)	147 (63)	0.287
Surgical approach (open)	1.8% (1)	2.1% (4)	1
Blood loss (ml)	50 (130)	50 (80)	0.901
Intraoperative blood transfusion (yes)	7.1% (4)	3.1% (6)	0.240
Intraoperative event (yes)	7.1% (4)	6.8% (13)	1
Stoma (yes)	3.6% (2)	5.7% (11)	0.738
Additional organ resection (yes)	26.8% (15)	22.6% (43)	0.591
Hyperglycemia (yes)	10.9% (6)	16.5% (30)	0.313
Hypothermia (yes)	66.1% (37)	50.5% (96)	0.040
Incorrect antibiotic prophylaxis (yes)	0	2.1% (4)	0.577
Vasopressors (yes)	48.2% (27)	72.4% (139)	** < 0.001**
Epidural (yes)	7.1% (4)	3.1% (6)	0.240

Categorical variables are expressed as proportions (%) and continuous variables as medians (interquartile range)

*p*-values < 0.05 were considered statistically significant and marked bold

After correction of preoperative anemia, changes in hemoglobin levels were significantly associated with surgery duration (155 versus 142 versus 155 min, *p* = 0.037) and vasopressor administration (76.9% versus 70.5% versus 54.5%, *p* = 0.010) (Table 5).

**Table 5:** Baseline characteristics: hemoglobin change after correction

	Unchanged (*n* = 143)	Declined (*n* = 13)	Increased (*n* = 156)	*p*-Value
Preoperative				
Age (years)	73 (18)	69 (19)	74 (15)	0.502
Sex (male)	53.1% (76)	76.9% (10)	48.7% (76)	0.137
ASA score	2 (1)	3 (1)	2 (1)	0.798
BMI (kg/m^2^)	25.4 (5)	23.8 (6)	25.4 (5)	0.703
Diabetes mellitus (yes)	17.3% (24)	15.4% (2)	21.2% (32)	0.672
Pathology (malign)	88.3% (113)	100% (11)	91.8% (135)	0.332
Neoadjuvant therapy (yes)	8.6% (11)	8.3% (1)	4.8% (7)	0.130
Intoxications				
Current smoker (yes)	6.3% (9)	15.4% (2)	7.1% (11)	0.138
> 14 packyears (yes)	42.1% (16)	40.0% (2)	42.6% (26)	0.993
Alcohol (yes)	43.5% (60)	41.7% (5)	50.0% (75)	0.508
> 4 units per day (yes)	1.8% (1)	0	1.5% (1)	0.918
Drugs (yes)	0	0	0	−
Perioperative				
Procedure				
Right-sided hemicolectomy	46.2% (66)	61.5% (8)	62.2% (97)	0.170
Extended right-sided hemicolectomy	4.2% (6)	0	5.1% (8)	
Ileocecal resection	3.5% (5)	0	0.6% (1)	
Transversum resection	4.9% (7)	0	2.6% (4)	
Left-sided hemicolectomy	10.5% (15)	7.7% (1)	5.1% (8)	
LAR/TME	7.7% (11)	7.7% (1)	7.1% (11)	
TaTME	0	7.7% (1)	0.6% (1)	
Sigmoidectomy	21.7% (31)	15.4% (2)	14.7% (23)	
Other	1.4% (2)	0	1.9% (3)	
Surgery duration (min)	150 (75)	155 (99)	142 (57)	**0.037**
Surgical approach (open)	1.4% (2)	0	2.6% (4)	0.594
Blood loss (ml)	50 (130)	100 (88)	50 (79)	0.592
Intraoperative blood transfusion (yes)	4.2% (6)	7.7% (1)	1.9% (3)	0.370
Intraoperative event (yes)	8.5% (12)	15.4% (2)	3.9% (6)	0.110
Stoma (yes)	2.8% (4)	7.7% (1)	5.1% (8)	0.487
Additional organ resection (yes)	23.6% (33)	15.4% (2)	23.2% (36)	0.797
Hyperglycemia (yes)	16.9% (23)	15.4% (2)	15.6% (23)	0.956
Hypothermia (yes)	60.1% (86)	30.8% (4)	50.6% (78)	0.058
Incorrect antibiotic prophylaxis (yes)	2.1% (3)	0	0.6% (1)	0.452
Vasopressors (yes)	54.5% (78)	76.9% (10)	70.5% (110)	**0.010**
Epidural (yes)	6.3% (9)	7.7% (1)	3.2% (5)	0.406

Categorical variables are expressed as proportions (%) and continuous variables as medians (interquartile range)

*p*-values < 0.05 were considered statistically significant and marked bold

### Secondary outcomes

CAL was observed in 6.1% (*n* = 53) of the patients. The severity of CAL was classified as ISREC grade A in 0%, B in 9.4% (*n* = 5), and C in 90.6% (*n* = 48). The incidence of CAL was not significantly associated with correction of preoperative anemia (*p* = 0.607) or with changes in hemoglobin levels after iron treatment (*p* = 0.736). Moreover, there was no significant relationship between the severity of CAL and preoperative correction or changes in hemoglobin levels (*p* = 0.318; *p* = 0.192).

The postoperative course is summarized in Table 6. The median length of stay was 4 days, which did not differ between patients with corrected and noncorrected preoperative anemia (*p* = 0.975) or changes in hemoglobin levels after treatment (*p* = 0.325). The incidence and severity of other postoperative complications did not differ significantly between the corrected and noncorrected group (*p* = 0.494; *p* = 0.235). Furthermore, changes in hemoglobin levels after correction were not associated with the occurrence or severity of postoperative complications (*p* = 0.776; *p* = 0.548). Intensive care unit stay (*p* = 0.058) and hospital readmissions (*p* = 0.488) also showed no significant differences between the corrected and noncorrected groups, nor were they related to hemoglobin levels after iron treatment (*p* = 0.177; *p* = 0.521). The mortality rate was 0.8% (*n* = 7) and was not significantly different between the corrected and noncorrected groups or changes in hemoglobin levels after iron treatment (*p* = 0.541; *p* = 0.838).

**Table 6:** Postoperative course

Correction of preoperative anemia			
	Not corrected (*n* = 56)	Corrected (*n* = 192)	*p*-value
CAL (yes)	3.6% (2)	5.3% (10)	0.607
Grade A	0	0	0.318
Grade B	50.0% (1)	10.0% (1)	
Grade C	50.0% (1)	90.0% (9)	
Other complications (yes)	23.2% (13)	19.0% (36)	0.494
Grade I	30.7% (4)	24.9% (9)	0.235
Grade II	38.5% (5)	47.2% (17)	
Grade IIIa	15.4% (2)	5.6% (2)	
Grade IIIb	0	13.9% (5)	
Grade IVa	7.7% (1)	0	
Grade IVb	0	2.8% (1)	
Grade V	7.7% (1)	5.6% (2)	
Length of stay (days)	4 (4)	4 (4)	0.975
Intensive care admission (yes)	12.5% (7)	4.7% (9)	0.058
Readmission (yes)	7.3% (4)	10.4% (20)	0.488
Death (yes)	1.8% (1)	1.1% (2)	0.541

Differences in hemoglobin level after correction				
	Unchanged (*n* = 143)	Declined (*n* = 13)	Increased (*n* = 156)	*p*-value
CAL (yes)	4.3% (6)	0	4.5% (7)	0.736
Grade A	0	0	0	0.192
Grade B	33.3% (2)	0	0	
Grade C	66.7% (4)	0	100% (7)	
Other complications (yes)	20.4% (29)	15.4% (2)	17.5% (27)	0.776
Grade I	28.0% (8)	0	33.4% (9)	0.548
Grade II	41.1% (12)	50.0% (1)	44.4% (12)	
Grade IIIa	10.3% (3)	0	7.4% (2)	
Grade IIIb	10.3% (3)	0	7.4% (2)	
Grade IVa	0	50.0% (1)	0	
Grade IVb	3.4% (1)	0	0	
Grade V	6.9% (2)	0	7.4% (2)	
Length of stay (days)	5 (3)	4 (4)	4 (3)	0.325
Intensive care admission (yes)	9.1% (13)	7.7% (1)	3.8% (6)	0.177
Readmission (yes)	9.2% (13)	0	8.3% (13)	0.521
Death (yes)	1.4% (2)	0	1.3% (2)	0.838

Categorical variables are expressed as proportions (%) and continuous variables as medians (interquartile range). *p*-Values < 0.05 were considered statistically significant

Alterations in the hemoglobin levels were classified as declined when levels dropped > 0.5 mmol/L, increased when elevated > 0.5 mmol/L and unchanged between −0.5 mmol/L and 0.5 mmol/L

CAL gradation according to ISREC; other complications gradation according to CD

### Regression analysis

Univariate regression analysis did not find a significant association between complications and anemia correction (*p* = 0.494). However, it did identify significant associations between complications and ASA score (OR 1.96, *p* = 0.011), surgery duration (OR 1.01, *p* < 0.001), and intraoperative events (OR 3.11, *p* = 0.018). Afterwards in multivariate regression analysis, this relationship was corrected for identified significant confounders in univariate analysis (Table 7). The multivariate regression analysis confirmed the absence of a significant association between complications and the correction of preoperative anemia (OR 0.90, 95% CI 0.41–1.96, *p* = 0.784).

**Table 7:** Regression analysis

	Univariate	*p*-value
	OR (95% CI)	
Age (years)	1.02 (0.99–1.04)	0.181
Sex (male)	1.22 (0.69–2.16)	0.494
ASA score	1.96 (1.17–3.29)	**0.011**
BMI (kg/m^2^)	0.95 (0.88–1.02)	0.121
Pathology (malign)	0.79 (0.30–2.05)	0.621
Procedure		0.283
Right-sided hemicolectomy	0.20 (0.04–1.02)	0.236
Extended right-sided hemicolectomy	0.85 (0.18–4.01)	0.839
Ileocecal resection	5.11 (0.98–26.61)	0.053
Transversum resection	2.92 (0.80–10.64)	0.105
Left-sided hemicolectomy	1.08 (0.34–3.40)	0.902
LAR/TME	1.08 (0.34–3.40)	0.902
TaTME	5.11 (0.31–84.09)	0.254
Sigmoidectomy	1.11 (0.50–2.46)	0.796
Other	5.11 (0.98–26.61)	0.053
Surgery duration (min)	1.01 (1.00–1.01)	**0.001**
Surgical approach (open)	0.86 (0.98–7.46)	0.887
Blood loss (ml)	1.00 (1.00–1.01)	0.755
Intraoperative blood transfusion (yes)	1.08 (0.22–5.20)	0.929
Intraoperative event (yes)	3.11 (1.21–8.00)	**0.018**
Hyperglycemia (yes)	0.94 (0.43–2.06)	0.872
Hypothermia (yes)	0.97 (0.55–1.72)	0.918
Incorrect antibiotic prophylaxis (yes)	1.44 (0.15–14.06)	0.756
Vasopressors (yes)	0.98 (0.54–1.76)	0.940
Epidural (yes)	2.25 (0.74–6.86)	0.153

	Univariate	*p*-value	Multivariate*	*p*-value
	OR (95% CI)		OR (95% CI)	
Not corrected preoperative anemia	1	0.494	1	0.784
Corrected preoperative anemia	0.78 (0.38–1.60)		0.90 (0.41–1.96)	

*p*-values < 0.05 were considered statistically significant and marked bold

^*^Corrected for ASA score, surgery duration, and intraoperative event

## Discussion

This study demonstrated a reduction in incidence and severity of perioperative anemia following the introduction of an enhanced care bundle. However, this reduction did not lead to a significant alteration in postoperative outcomes. The enhanced care bundle facilitated early and consistent detection and treatment of preoperative anemia in patients undergoing elective colorectal surgery. In recent years, the significant impact of preoperative anemia on postoperative complications has become increasingly evident. As a result, anemia management has gained prominence in prehabilitation and other preoperative optimization care bundles aimed at reducing overall morbidity. However, this approach had not previously been consistently integrated into colorectal care pathways, and the DoubleCheck study was the first to achieve this in a prospective multicenter trial [13]. This prospective subanalysis provided valuable insights into the role of preoperative anemia correction on perioperative and postoperative outcomes.

This study did not demonstrate a significant association between postoperative complications and the correction of preoperative anemia, nor with changes in hemoglobin levels following correction. This outcome may be attributed to the study’s focus on the effect of anemia correction alone, despite the fact that the overall intervention encompassed six distinct subinterventions. The preventive impact of each individual subintervention on complications may be too subtle to detect when examined individually. Furthermore, optimizing multiple factors simultaneously may have a synergistic effect on the enhancement of the perioperative condition. Both outcomes indicate that anemia may be more accurately regarded as an indicator of overall poor physiological fitness rather than as an isolated risk factor, and serves as a warning for potential poor postoperative outcomes. Therefore, routine preoperative administration of iron alone, without concurrently optimizing other CAL risk factors, is not recommended.

Our findings revealed a significant association between perioperative hyperglycemia and preoperative anemia (*p* < 0.001). This observation may be partially explained by the higher prevalence of diabetes mellitus in patients with preoperative anemia (7.8% versus 16.4%, *p* < 0.001). However, the association remained significant in the subgroup analysis of the patients without diagnosed diabetes (Table 8). The relationship between anemia and hyperglycemia has been established previously, with proposed explanations suggesting low hemoglobin levels are linked to impaired glucose hemostasis and poor glycemic control [21, 22]. Moreover, both diabetes mellitus type 1 and 2 are known to exacerbate anemia. These interrelated factors underline the importance of simultaneously optimizing all risk factors for CAL.

**Table 8:** Subgroup analysis perioperative hyperglycemia

Diabetes mellitus			No diabetes mellitus		
	Anemia	No anemia		Anemia	No anemia
Hyperglycemia	46.4% (26)	50.0% (22)	Hyperglycemia	9.4% (22)	4.0% (19)
No hyperglycemia	53.6% (30)	50.0% (22)	No hyperglycemia	90.6% (213)	96.0% (459)
		*p* = 0.723			***p* = 0.004**

Variables are expressed as proportions (%)

*p*-values < 0.05 were considered statistically significant and marked bold

The definition of anemia and established hemoglobin cutoff values remain a topic of ongoing debate. According to the World Health Organization, anemia is defined as “a condition in which the number of red blood cells or the hemoglobin concentration within them is lower than normal (< 13.0 g/dL in male, < 12.0 g/dL in female)” [23]. Commonly used cutoff values for hemoglobin levels vary between 7.0 mmol/L and 8.5 mmol/L, with some studies employing gender-specific thresholds. For instance, Beutler et al. (2006) identified cutoff levels of 8.5 mmol/L (13.7 g/dL) in male patients and 7.6 mmol/L (12.2 g/dL) in female patients in a comprehensive literature review [24]. However, more recent studies suggested a lower cutoff value of 7.4 mmol/L (12.0 g/dL), while others propose optimal cutoff values of 8.4 mmol/L (13.5 g/dL) in male patients and 7.4 mmol/L (12.0 g/dL) in female patients [25, 26]. This lack of uniformity seems to depend on the specific investigated study population. Nevertheless, it is generally agreed that the cutoff values should differ for male individuals and female individuals, with male individuals having higher thresholds, typically within the range of 7.5 mmol/L and 8.5 mmol/L.

Furthermore, although intravenous iron transfusion in generally found to be more effective than oral supplementation, practice variations still frequently occur in daily practice [9, 27]. This variation may be attributable to the difference in costs, with intravenous transfusions being approximately six times more expensive [28]. Given the limited time between diagnosis and surgery, oral suppletion may not elevate hemoglobin levels rapidly enough, leading to preference for intravenous administration. In addition, colorectal cancer is associated with alterations in iron absorption, which can impair the efficacy of oral supplements [29]. Therefore, intravenous supplementation is particularly advantageous for oncological patients, who have a higher prevalence of preoperative anemia (81.9% versus 90.7%, *p* < 0.001). While the difference between intravenous and oral treatment has been less thoroughly studied in patients with benign surgical indications, intravenous transfusions also appear to be the preferable option in this subgroup [30]. It was decided to avoid a rigid protocol regarding the administration method for iron therapy (oral versus intravenous) and the exact timing of hemoglobin re-measurement. It was hypothesized that the current approach would improve adherence to preoperative anemia correction.

A strength of this study is its international, multicenter, and prospective design. Additionally, the proposed approach for anemia detection and correction involved widely accessible, simple, and cost-effective interventions, making it feasible for global implementation in colorectal care pathways. However, the study had some limitations, including the potential confounding effects of other safety procedures aimed at reducing complications. Nonetheless, we believe these factors had minimal impact on hemoglobin levels and the availability of optimization resources. Secondly, a diverse patient cohort was intentionally investigated, as we believed that preoperative optimization has the potential to benefit all individuals undergoing colorectal surgery. Additionally, the variance in lymphadenectomies among patients was not reported, and therefore not investigated.

In conclusion, this study demonstrated that early detection and correction of preoperative anemia is achievable with the implementation of the DoubleCheck enhanced care bundle, but does not change postoperative outcomes. Preoperative anemia may be more indicative of overall poor physiological fitness rather than an isolated factor. Implementation of early and consistent preoperative anemia detection and correction will enhance the quality of care for patients undergoing colorectal surgery.

## Data Availability

Deidentified individual participant data collected in this study and additional relevant related documents can be made available upon request by contacting A.d.W. (a.dewit1@amsterdamumc.nl) or F.D. (f.daams@amsterdamumc.nl). There are no date restrictions on the availability of the data. The investigators will be allowed to approve all research conducted with the shared data.
